# Dengue Vaccines: Strongly Sought but Not a Reality Just Yet

**DOI:** 10.1371/journal.ppat.1003551

**Published:** 2013-10-03

**Authors:** Rosa María del Angel, Jorge Reyes-del Valle

**Affiliations:** 1 Departamento de Infectómica y Patogénesis Molecular, CINVESTAV-IPN, Mexico City, México; 2 School of Life Sciences, Arizona State University, Tempe, Arizona, United States of America; University of Notre Dame, United States of America

## Dengue Is a Serious Public Health Problem

Dengue virus (DV) infections cause undisputedly the most important arthropod-borne viral disease in terms of worldwide prevalence, human suffering, and cost. Worldwide DV infection prevalence in 2010 was between 284 to 528 million cases [1]. Approximately 84% of these cases come from Asia and the Americas, where the cost for emerging economies can be as high as 580 million dollars per year [2]. Thus, the need for an efficient vaccine against DV is extreme.

While up to 90% of dengue cases are either asymptomatic or cause an underreported, self-limited, flu-like illness, symptomatic DV infection manifests as two main clinical forms: 1) dengue fever (DF) and 2) severe dengue (SD). DF symptoms include high-degree fever, headache, myalgia with arthralgia, retro-orbital pain, and rash. SD evolves clinically as a life-threatening complication from DF. SD is characterized by plasma leakage, hemoconcentration, hemorrhagic shock, and organ failure, which may lead to patient death. Any of the four described DV serotypes can be responsible for either DF or SD, although the precise mechanisms leading to SD are still unclear. Evidence suggests factors such as viral virulence, host gene background, and secondary DV infections contribute to the exacerbation of the disease. In consequence, preventive strategies through vaccination must consider tetravalent formulations.

## Dengue Virus Is a Flavivirus

**Figure 1:**
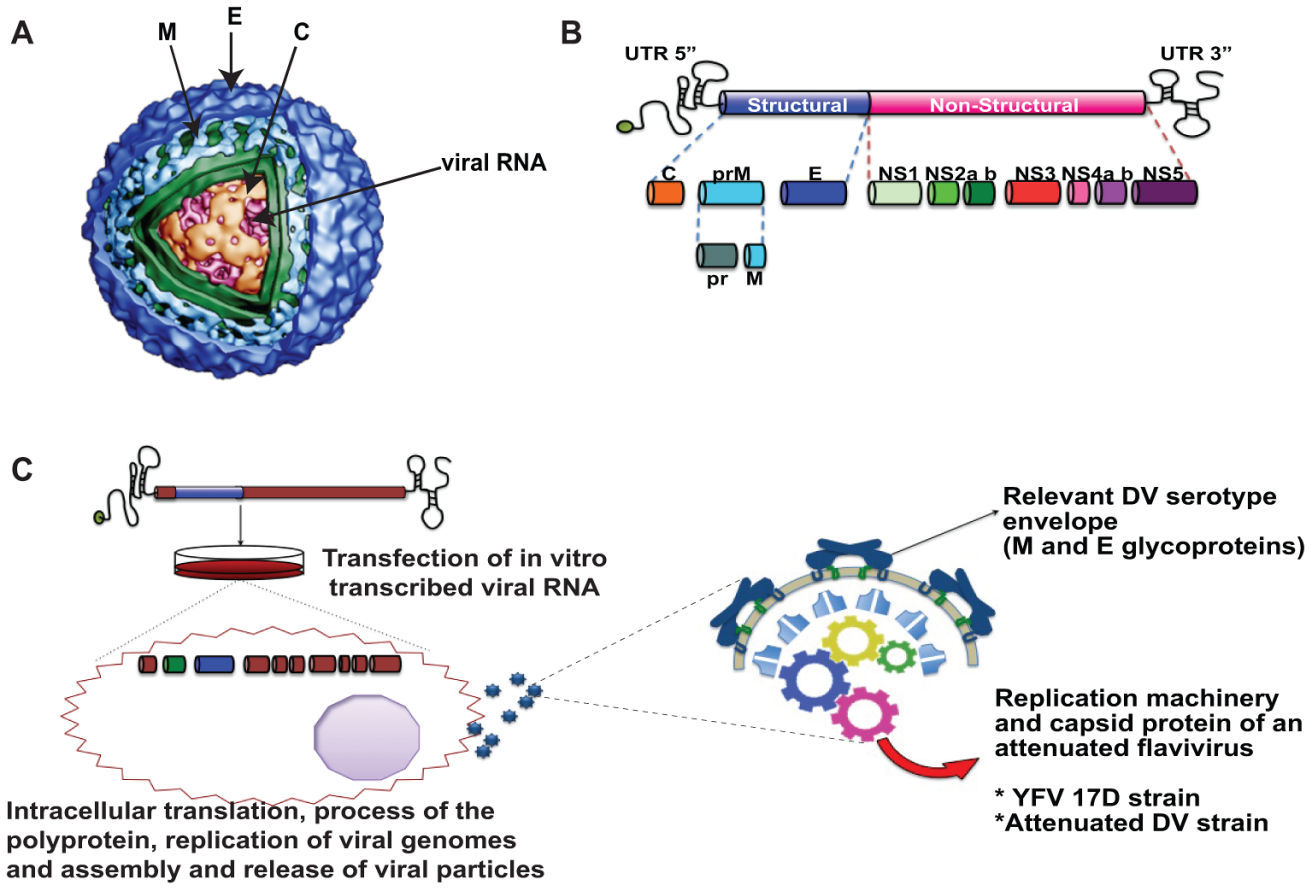
Dengue virus structure. **A.** DV virions are enveloped, containing three structural proteins; the envelope (E) and membrane (M) proteins (dark and light blue, respectively) are anchored to the viral membrane (green), and the capsid protein (C) (orange) covers the viral genome (red) (kindly provided by Dr. Richard J. Kuhn). **B.** The dengue genome is a single-stranded, positive-polarity RNA with a unique open reading frame flanked by 5′ and 3′ untranslated regions (UTR). It encodes for three structural and seven nonstructural proteins, represented by colored, stylized boxes. The name of DV viral proteins is indicated. **C.** Diagram of chimeric DV vaccine approach. Chimeric flaviviruses are constructed by swapping the prM and E coding sequences from an attenuated backbone for those of a heterologous DV strain. Viral RNA is obtained in vitro and transfected. Vero cells are used to amplify the infectivity of the chimeric virus, which is formed by the respective DV serotype envelope proteins with the replication machinery (represented by gears) of an attenuated, related flavivirus.

The *Flaviridae* family, of which DV is a member, consists of enveloped, positive-stranded RNA viruses. The flavivirus genome encodes a polyprotein, which is processed by cellular and viral-encoded proteases into ten proteins (Figure 1A–B). The virion core contains the viral genome encased in the capsid (C) protein. The virion membrane contains two virus-coded type I membrane glycoproteins. The larger envelope (E) protein is essential for viral attachment and entry and is also the main target of neutralizing antibodies. The membrane protein (M) is synthesized as a precursor protein (prM) that is processed during export; it prevents E conformational changes from occurring during viral assembly. Besides these three structural proteins (C-prM-E), the viral genome codes for seven nonstructural proteins important for viral replication, translation, and the proteolytic processing of the polyprotein.

Since E is exposed on the virion surface, it contains the epitopes recognized by neutralizing antibodies and its gene is responsible for the genetic variation resulting in four DV serotypes [3]. The mature flavivirus virion particle has a herringbone array of 90 tightly packed E glycoprotein dimers parallel to the virion surface. Each 500-amino-acid E monomer contains a 400-amino-acid N-terminal ectodomain that folds into three structural domains. It has been posited that antibodies directed against domain III are of highly specific neutralizing capacity, while antibodies directed against the other two domains, neutralizing to some extent, can cross-react with other DV serotypes.

## The Ideal DENV Vaccine

Vaccination has been the most desired strategy for controlling the spread of DV. Neutralizing antibodies directed against mosquito-borne flavivirus envelopes can prevent the development of infectious disease. This has been beautifully illustrated by the development of successful vaccines against other related mosquito-borne flaviviruses with similar structure, specifically the attenuated strain 17D vaccine against yellow fever virus (YFV) and the attenuated strain 14-14-2 against Japanese encephalitis virus (JEV), both obtained by serial passage in cell culture. Importantly, for both vaccines, a neutralization titer of at least 1∶10 is protective. Thus, the most useful and documented correlate of protection for flaviviruses is the presence of neutralizing antibodies assayed by the plaque reduction neutralization assay (PRNA).

Why then has the development of a DV vaccine proven so challenging? Natural DV infection triggers a robust, neutralizing immunity that provides an apparently life-long protection against the infecting DV serotype and a short-lived (months) cross-protection against heterologous DV serotypes. Interestingly, the humoral response to DV not only mediates protection though viral neutralization, but also seems to play a major role in the development of more severe forms of dengue disease. Dengue hemorrhagic fever and dengue shock syndrome (DHF/DSS) cases are often associated with secondary DV infections with a heterologous DV serotype. Subneutralizing, heterotypic antibodies induced during a primary infection with one serotype of DV form complexes with a different serotype of DV during secondary infection, enhancing uptake of infectious particles by Fc-receptor–bearing permissive cells. This phenomenon, called antibody-dependent enhancement (ADE), results in an increased number of infected cells and a higher viremia that, combined with a pro-inflammatory cytokine response from an enhanced infection of macrophage and dendritic cells, have been related with SD [4]. Furthermore, antibodies to the precursor form of M, the prM protein, can also induce ADE [5]. Since an important fraction of viral particles in patients’ blood are “immature,” displaying prM on their surface, these may become fully infective in the presence of anti-prM antibodies. Thus, ADE reinforces the necessity for a tetravalent DV vaccine preparation, a requirement that has been a significant obstacle to successful vaccine development. The realistic approach toward this goal has consisted of developing monovalent vaccines capable of inducing homotypic protection, and subsequently combining them into a tetravalent formulation. However, the induction of a strong and balanced protective immunity against the four DV serotypes to avoid the potential risk of ADE after vaccination has been challenging. Several DV vaccines are currently in different stages of development; due to space constraints, we will refer to those of significant interest.

## Attenuated and Recombinant DV Vaccine Candidates

Attenuating DV is a quest that has lasted more than 60 years [6]. Classic attenuation by serial passage in cell lines different from the traditional animal host has been achieved for DV. The balance between safe attenuation and robust immunogenicity is achieved differently for each DV serotype. In particular for DV2, after 53 passages in primary dog kidney cells (PDK53) the virus lost its capability to induce viremia in nonhuman primates, while stimulating robust neutralizing immune responses [7]. After almost two decades of experimentation we have corroborated that reactogenicity is associated with an insufficient passage history, learned that dose components and the time between multiple doses are of paramount importance to obtain balanced immunity against the four serotypes, and that a formulation that causes fever in adults is probably safe to use in children. Recently, a phase II clinical trial in 86 adult volunteers documented safety and an acceptable immunogenicity that was higher in individuals with prior DV or other flavivirus contact history [8].

As an alternative to attenuation by serial passages, DV can also be attenuated by targeted mutagenesis. Deletion of 30 nucleotides (Δ30) in the 3’ end of the DV1 and DV4 RNA genome results in a reduction of viral replication fitness while maintaining immunogenicity [9]. Swapping the entire 3’ nontranslated region of DV3 with that of DV4 Δ30 attenuated the virus. DV2 was attenuated by chimerization using the replication machinery of DV4 Δ30. A phase I clinical trial of tetravalent vaccine formulations (TetraVax-DV) using these immunogens was conducted in flavivirus-naïve adults and reported recently [10]. Close to 90% seropositivity to all serotypes except DV2 was obtained after a single dose. Viremia after vaccination correlated with a trivalent immunity.

A major breakthrough in DV vaccine research came with the demonstration that mosquito-borne flaviviruses may replicate as chimeric viruses in which the envelope genes M and E of an attenuated vector genome are replaced with the cognate genes of the target virus. This strategy, depicted in Figure 1C, has resulted in two different types of chimeras important in DV-attenuated vaccines:

- Using the replication machinery of the YFV 17D vaccine strain, Chimerivax displays each of the four DV serotype glycoproteins in a separate functional viral particle. It constitutes the most advanced vaccine candidate to be tested in humans so far. This vaccine showed promising results in phase I clinical trials in endemic and nonendemic countries. However a phase IIb clinical trial in 2,669 Thai schoolchildren with a tetravalent vaccine showed poor efficacy (30.3%, 95% CI - 13.4 to 56.6) of protection against febrile DV infections [11]. Subjects received three doses at zero, six, and 12 months and were monitored for up to two years after the first dose. While one or more doses of the vaccine reduced incidence of DV1, DV3, and DV4 by 90%, an efficacy of only 9.2% (95% CI -75.0 to 51.3) was observed for DV2, which negatively impacted the overall protection. Regrettably, fewer than 5% of vaccinees and control subjects were studied serologically for DV neutralization after completion of the vaccine schedule. Interestingly, after two years of incomplete protection against the four serotypes no enhanced DV infectious disease was documented, which suggests that trivalent immunity may prevent the development of SD and underscores the need to revisit ADE theoretical risk among vaccinees.
- The other chimeric construction approach uses the replication machinery of the aforementioned attenuated DV2 PDK53 vaccine candidate. Again, the relevant DV envelope glycoproteins are displayed on the surface of the chimeric particle. The tetravalent formulation (DENvax) is currently in a dose-escalating, phase I clinical trial (*ClinicalTrial.gov* identifier NCT01224639).

## Inactivated and Subunit Vaccines

There are two successful examples of inactivated vaccines against related arthropod-borne flaviviruses, tick-borne encephalitis, and JEV. This strategy is appealing for the pursuit of a DV vaccine because an inactivated vaccine is unlikely to elicit imbalanced immune responses stemming from infection interference. Protective immune responses in macaques after three doses of a concentrated, formalin-inactivated, alum-adjuvanted DV2 vaccine (PIV for purified, inactivated vaccine) candidate have been documented [12]. Furthermore, PIV immunogenicity and protective ability when administered in complex with modern, proprietary AS05 and AS08 adjuvants was comparable to a single dose of corresponding live-attenuated vaccine [13].

Subunit vaccines have also been explored. Immunization with an insect-expressed E protein ectodomain from DV2 is capable of inducing protection in macaques [14]. Once again, the use of adjuvants seems important to achieve robust immunity via this mechanism. A phase I clinical trial, designed to assess the safety of alum-adjuvanted DV1 E antigen, in sixteen adult volunteers, has been concluded and is pending publication of results (*ClinicalTrial.gov* identifier NCT00936429).

In summary, the strength and longevity of neutralizing immune responses elicited by attenuated vaccines outperforms responses observed with replication-defective approaches. Still, safety and the possibility of combining vaccine components without the possibility of viral interference adds enthusiasm for the latter approach. Interestingly, the potential for a prime-boost schedule was assessed in primate model [15]. In this report, macaques received a single dose of a tetravalent PIV vaccine and then a derivative, tetravalent, attenuated formulation two months later. All of the animals developed a solid immunity against the four DV serotypes documented by the absence of viremia following a wild-type challenge six months after the last vaccine dose.

## Challenges in the Quest for a Dengue Vaccine

There are two main windows of opportunity to build upon toward realizing efficient vaccination for DV. First, a clinically relevant animal model for dengue infection and vaccine development is lacking. Rhesus monkeys do not show clinical signs of infection after a wild-type DV challenge; instead the intensity and length of viremia serves as a proxy to infer protection. Second, rigorous correlates of protection have not been established for DV. The best available indicator of immunogenicity is the titration of neutralizing antibodies, however titration of plaque reduction neutralization antibodies has not been promoted to a *bona fide* correlate of protection because of ambiguous results pertaining to the protective titer. Testing the protection efficiency of tetravalent vaccine candidates in volunteers may answer both questions. Results from the first human DV challenge experiments have been recently published and demonstrate the viability of this approach [16]. Paraphrasing Vesalius, DV vaccine development may have been “deceived by monkeys” but is on the right path to become a huge benefit for mankind.
